# Transoral Robotic Surgery: Step-by-Step Radical Tonsillectomy

**DOI:** 10.1155/2014/497528

**Published:** 2014-04-06

**Authors:** Jose Granell, Ivan Mendez-Benegassi, Teresa Millas, Laura Garrido, Raimundo Gutierrez-Fonseca

**Affiliations:** Otorhinolaryngology Department, Rey Juan Carlos University Hospital, Gladiolo s/n, Mostoles, 28933 Madrid, Spain

## Abstract

*Introduction*. Transoral robotic surgery (TORS) radical tonsillectomy is an emerging minimally invasive surgical procedure for the treatment of cancer of the tonsil. The detailed surgical technique and claims for its reproducibility have been previously published. *Case Presentation*. We present a patient with a T2N2bM0 epidermoid carcinoma of the tonsil to illustrate step by step the surgical procedure for TORS radical tonsillectomy. Neck dissection and TORS were staged. No surgical reconstruction of the defect was required. No tracheostomy was necessary. The patient could eat without any feeding tube and was on full oral diet on the fifth postoperative day. *Discussion*. The transoral approach offers the benefits of minimally invasive surgery to patients with cancer of the tonsil. The excellent exposure and high precision provided by robotic instrumentation allow the surgeon to closely follow and accomplish the surgical steps, which is the best warranty for safety and effectiveness.

## 1. Introduction

Weinstein et al. described transoral robotic surgery (TORS) at the Hospital of the University of Pennsylvania, Philadelphia, following a systematic basic and clinical investigation that started in 2004 [1]. They also led the first training program which established the core for the subsequent development of TORS in USA and worldwide. TORS is based on the application of the* da Vinci* surgical system (Intuitive Surgical Inc., Sunnyvale, CA) for transoral approaches. As the* da Vinci* was not designed to be used transorally, some basic modifications had to be done (e.g., in the operating room setup and in the devices for transoral exposure); subsequently, every transoral procedure had to be readapted to the new technology.

Although TORS has greatly expanded since FDA clearance in December 2009, it is still an emerging procedure. Therefore, many of the centres are just starting or still in their learning curve, which is considered to be quite short, particularly for surgeons with previous experience in endoscopic surgery [2]. The anatomical area that gets more benefit from TORS is the oropharynx. Among the many applications that have been assayed, TORS has been found to be particularly useful for the treatment of cancer of the oropharynx, where it has chances to become the treatment of choice [3].

Differently from plain tonsillectomy in which dissection is carried through the peritonsillar space to excise the contents of the tonsillar fossa (i.e., the tonsil), radical tonsillectomy includes the resection of the walls of the tonsillar fossa. Dissection is done laterally to the constrictor muscle, into the parapharyngeal space, which provides the surgical margin required for oncologic safety. Weinstein et al. described TORS radical tonsillectomy [4] based on a previous transoral nonrobotic radical tonsillectomy (lateral oropharyngectomy) technique [5], whose description was originally made by the French surgeon Huet in 1951 [6]. The authors underlined that the unparalleled vision and dexterity offered by the robotic instrumentation allowed the procedure to be highly effective and reproducible [7].

As for every surgical technique, safety and effectiveness are based on adequate indication and skilled performance. Sound knowledge of the technique and laboratory training are the foundations for success. There are not many published descriptions of the technique for TORS radical tonsillectomy, except for the original ones; therefore, we found that a detailed description of the surgical steps could be useful and further support reproducibility. As we were privileged to learn the technique from the original authors we also thought we could offer some practical tips. Therefore we set to present the step-by-step technique of TORS radical tonsillectomy with a real surgical case.

## 2. Case Presentation

A male patient, aged 66, was diagnosed of a poorly differentiated epidermoid carcinoma of the right tonsil extending to the anterior tonsillar pillar and with limited extension to the tongue base. After radiologic evaluation with cervical CT and PET-CT it was staged as T2N2bM0.

The Institutional Head and Neck Cancer Committee advised surgical treatment. Surgery was staged to minimize morbidity. Right functional neck dissection was performed first, followed by an approach to the primary tumour two weeks later.

**Figure 1:**
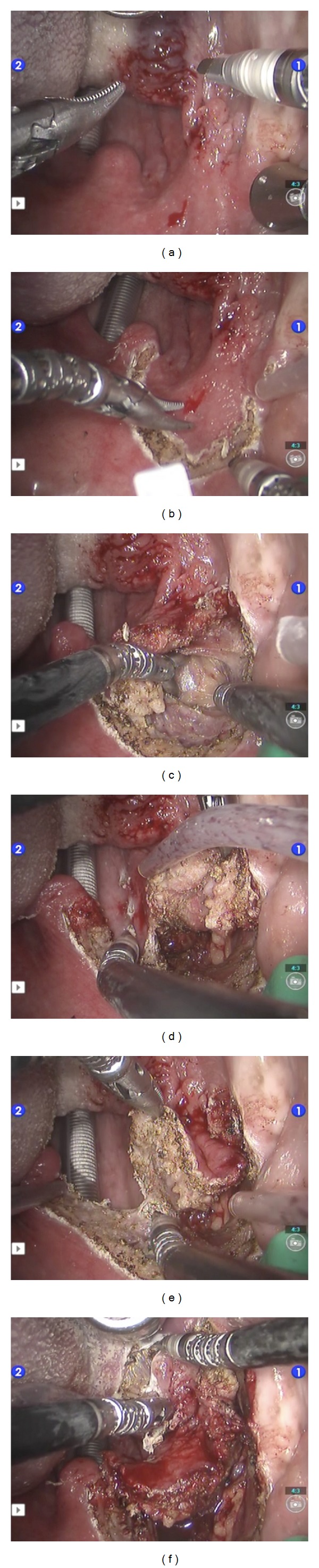
TORS radical tonsillectomy intraoperative views. Right hand: spatula tip monopolar cautery. Left hand: Maryland dissector (left-right, top-down). (a) Right tonsil epidermoid carcinoma extending to the anterior tonsillar pillar and tongue base. (b) Mucosal incision with a wide arch in the soft palate to fall laterally to the constrictor muscle. (c) Lateral limit: dissection of the parapharyngeal fat, which is pushed laterally off the constrictor. (d) Medial limit: posterior pharyngeal wall. (e) Superior limit: soft palate cut. (f) Inferior limit: tongue base cut.

The patient was scheduled for TORS radical right tonsillectomy (Figure 1). A* da Vinci* S HD surgical system was used. The patient was put under general anaesthesia with nasotracheal intubation. A retraction suture was placed on the tip of the tongue to help placing it while exposing the surgical field. The oral cavity and pharynx were exposed with a Crow-Davis mouth gag with a Russel-Davis blade. The mouth gag was doubly stabilized with the aid of an articulated scope holding arm (Karl Storz 28272HC). Patient side cart of the* da Vinci* was docked by the right patient side. The 0° double camera was loaded in the endoscope camera manipulator arm and the 5 mm Endowrist instruments in the patient side manipulator arms 1 and 2, through 5 mm flared cannulas (Intuitive 420262). As it is desirable to pull towards de midline, the Maryland dissector (Intuitive 420143) is used in arm 2 (contralateral side of the lesion), controlled by the left master tool manipulator (MTM), and the spatula tip monopolar cautery (Intuitive 420142 with disposable tip 400160) in arm 1 (right MTM, ipsilateral side). All the basic TORS procedures are described with exclusive instrumentation with Maryland dissector and monopolar cautery and it should be exceptional to need other tools. The bedside assistant used a couple of* baby*-Yankauer Suction Cannulas.

**Figure 2:**
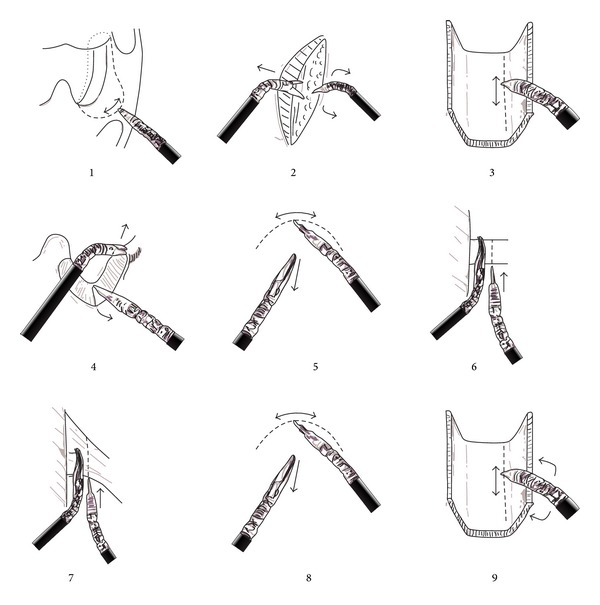
Schemes of the surgical steps for transoral robotic radical tonsillectomy.  Step 1. Initial incision to mark the superior and lateral superficial limits (dashed line). The incision is made with the monopolar cautery in a* question-mark* fashion to extend into the pterygomandibular raphe. The dotted line shows the inferior and medial superficial limits that will be marked later.  Step 2. Deep dissection starts after transecting the superior constrictor muscle. The parapharyngeal fat pad is reached and bluntly dissected laterally.  Step 3. An index cut in the posterior pharyngeal mucosa marks the superficial medial limit.  Step 4. The superior limit is completed with full width transection of the tonsillar pillars.  Step 5. Inferior superficial limit in the tongue base. Steps 6 and 7. Completion of the lateral limit by transection of the styloglossus (6) and stylopharyngeus muscles and the rest of connective tissue attachments (7).  Step 8. Completion of the inferior limit (deep tongue base).  Step 9. Completion of the deep medial limit (superior constrictor muscle) approaching either medially, laterally, superiorly, or inferiorly depending on the exposure.

Surgical steps are detailed (Figure 2).

**Step 1 (mucosal incision in the soft palate)**

Mucosal incision is outlined with the cautery. It runs from the free edge of the soft palate, lateral to the uvula at the point where tonsillar pillars meet and down the anterior tonsillar pillar. It is designed in a* question-mark* fashion, extending more laterally in the superior portion. This curvature assures complete inclusion of the cupula of the tonsil, entering into the right dissection plane laterally. Dissection of the deep planes starts precisely at the most superior and lateral point: superior constrictor muscle is exposed and transected to find a dissection plane just lateral to it.

**Step 2 (dissection of the superior constrictor muscle from parapharyngeal fat)**

The constrictor muscle is bluntly dissected from the parapharyngeal fat pad with the Maryland with the jaws opened holding the constrictor medially and the spatula, used as a blunt dissection tool, pushing laterally. Dissection is carried down to the level of the styloglossus muscle. Medial pterygoid muscle and mandible will be found in the superior-lateral edge of the dissection. The pulse of the internal carotid artery can be intuited under the parapharyngeal fat, but the carotid needs not to be exposed. Mucosal incision can then be extended downwards into the anterior tonsillar pillar and medially into the soft palate.

**Step 3 (posterior mucosa index cut)**

At this point an index cut is done vertically in the posterior pharyngeal mucosa to mark the medial limit of the resection. It is done just as deep as the mucosa and care is taken to avoid cutting the constrictor muscle at this moment. This area will be the last to be cut before taking out the specimen, but as dissection usually will come lateral to medial there is a risk of inadvertently taking more amount than the desired of posterior pharyngeal mucosa, leading to unnecessary morbidity.

**Step 4 (superomedial cut)**

A straight perpendicular cut is done through the whole depth of palatoglossus and palatopharyngeus muscles (tonsillar pillars), all the way down to the prevertebral fascia. Prevertebral fascia needs to be exposed and constrictor muscle bluntly dissected from it.

**Step 5 (tongue base cut)**

At the inferior edge of the resection, about 1 cm of tongue base muscle is included in the resection. This will assure a safe inferior margin in a standard resection, but the amount of tongue base muscle can be increased depending on the extension of the tumour (like in this case). Dissection is carried out just as deep as that in the parapharyngeal area (at the level of the styloglossus muscle); to avoid damaging branches of the lingual artery before adequate exposure to control any eventual bleeding is warranted.

**Step 6 (styloglossus cut)**

Styloglossus muscle is encountered crossing lateral to medial in the parapharyngeal dissection. It is dissected, completely individualized, and then grasped with the Maryland by the constrictor muscle and cut under direct vision with the Bowie, just lateral to the Maryland. Avoiding a far lateral incision will protect the carotid system from damage, while the maneuver with the Maryland will ensure about 1 cm oncological margin from the constrictor. The rationale for this step is that, eventually, styloglossus could be pulled laterally “off the constrictor” before cutting it, and the cut could be done too close. There is a dehiscence of the buccopharyngeal fascia at the point where the styloglossus meets the constrictor, which could risk a positive margin.

**Step 7 (stylopharyngeus cut)**

**Figure 3:**
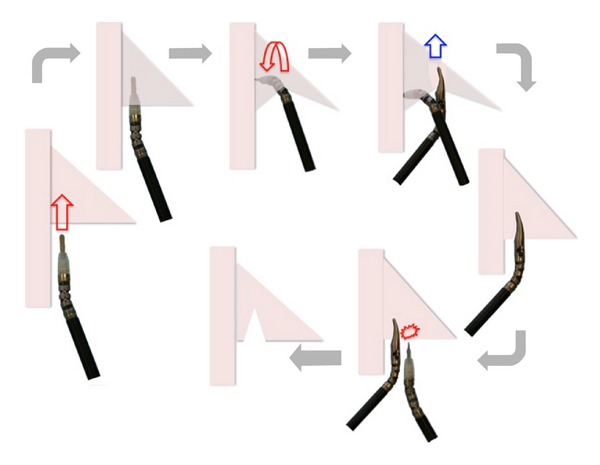
Stylopharyngeus cut sequence (from left, clockwise). The quadrangular shape represents the superior constrictor muscle and the triangular one the stylopharyngeus, right side. The aim is to completely cut the stylopharyngeus about 1 cm lateral to the constrictor. First the Bowie is inserted dissecting between the prevertebral fascia and the stylopharyngeus muscle, parallel to the constrictor. With the tip of the Bowie resting on the fascia, the* wrist* of the instrument is turned upwards. This creates a space under the muscle. The Maryland is crossed under the Bowie to grasp the muscle with the jaws parallel to the constrictor. Finally, the Bowie is used to cut the muscle just lateral to the Maryland. The sequence can be repeated as many times as necessary to completely split the stylopharyngeus.

A similar procedure is described for the same reasons to cut the stylopharyngeus muscle. However, unlike the styloglossus, stylopharyngeus muscle and the remaining tissue attachments to the constrictor appear in a* fan-like* fashion and therefore cannot be cut in one step. An original and ingenious sequential maneuver was described to safely accomplish the final lateral cut with both “hands” (Figure 3). The specimen is then free from the lateral attachments.

**Step 8**

Finish the tongue base cut.

**Step 9 (posterior wall cut)**

The constrictor muscle is cut at the medial deep limit of the resection, at the transition from the lateral to the posterior wall of the oropharynx.

Specimen is then completely free and can be taken out of the surgical field by the assistant. A couple of sutures can be done from the remaining posterior pharyngeal wall to the soft palate, to help nasopharyngeal closure when swallowing. The rest of the wound is left open; a hemostatic agent can be applied topically (we used none). No reconstruction was required. Intraoperative margins were free of disease. Definitive surgical margins were free of disease.

When managing the specimen, care is taken not to grab the mucosal margins, to avoid creating artifacts that could disturb pathological analysis. Specimen is obtained in a single block. To manage intraoperative bleeding, either plain monopolar cauterization or vascular clips may be used. Also bipolar forceps could be used by the beside assistant. No mayor vascular structures requiring clips were found in this case. Glossopharyngeal nerve is usually cut, but patients do not have specific complains.

The patient was kept under orotracheal intubation in the intensive care unit for 24 hours, and afterwards the tube was removed. No tracheostomy was necessary. Oral diet was started on postoperative day 3, and patient was on full oral diet on day 5. Pathologic study confirmed neck staging as pN2b; therefore, postoperative external radiation (IMRT) was indicated. The patient developed grade 2 mucositis and grade 2 dysphagia, but he needed no feeding tube at any point of the treatment.

**Figure 4:**
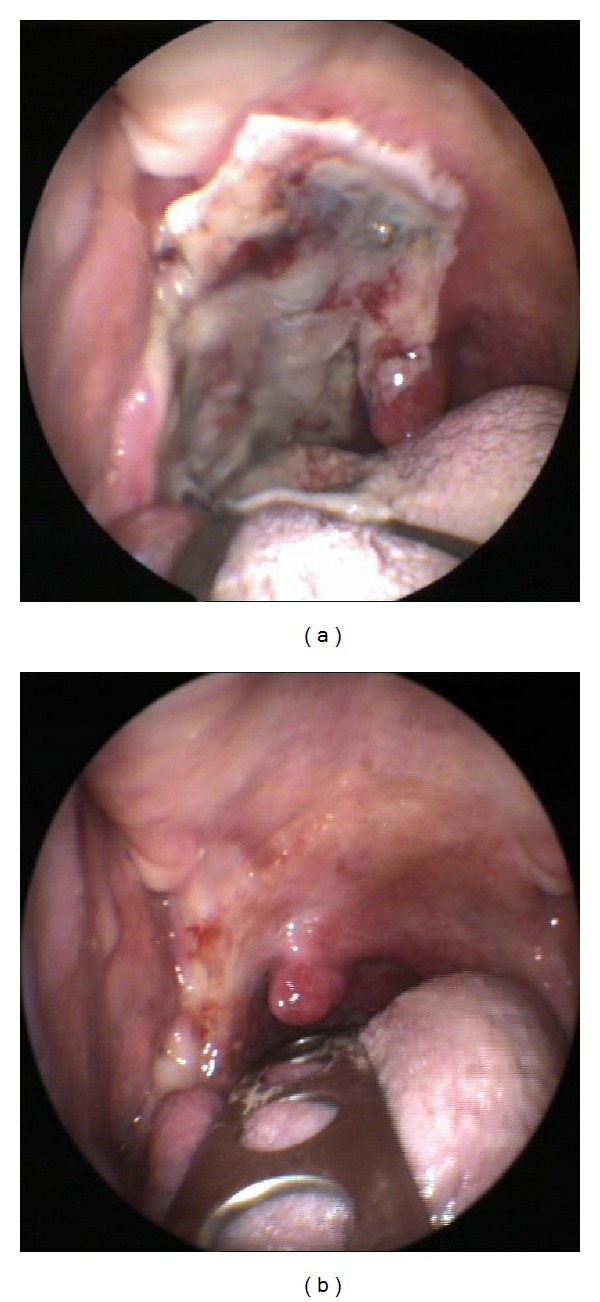
Postoperative anterior pharyngoscopy. (a) Immediate surgical scar at 5th postoperative day. Patient was on full oral diet. (b) Late scar. Note the marked lateralization of the uvula due to the contraction of the scar.

Posttreatment examination revealed the expected lateralization of the remaining oropharyngeal tissues, with the uvula appearing frankly right sided (Figure 4). Swallowing is normal. Speech is normal.

## 3. Discussion

The aim of the surgical treatment of cancer is to completely excise the tumour with adequate safety margins. This is irrespective of the surgical technique (which is usually defined by the approach). All surgical approaches are designed to minimize the damage associated with the approach itself. This motto is taken to its maximum in what is called minimally invasive surgery (with* minimally invasive* actually not referring to the* surgery* but to the* approach*). Transoral approach is the minimally invasive approach to the oropharynx.

Our standard surgical approach to this patient's tumour would have been a one-stage surgery including tracheostomy, neck dissection, paramedian mandibulotomy, lateral oropharyngectomy, and reconstruction with a fasciocutaneous anterolateral thigh free flap. It is important to remark that the surgical goal of this procedure would have been to obtain the same surgical specimen as the one obtained transorally and also to note that the need for and the type of reconstruction do not depend on the surgical specimen but on the surgical damage, which includes the surgical approach and the surgical excision. Also, there is an added benefit of secondary healing in the oropharynx. The scar will contract, partially closing the gap of the excised portion of the constrictor muscle (which will typically appear as a lateralization of the uvula towards the operated side). The contracted scar will perform better than noncontractile flaps in the pharyngeal phase of swallowing [8].

The rationale for the approach is oncologic and surgical safety. Transoral approach for radical tonsillectomy with conventional nonrobotic instrumentation is of course possible (actually, as remarked, TORS technique was described based on a modification of a previous technique). But many surgeons would not feel that transoral conventional approach will fulfil the safety requirements (both oncologic and surgical) and would opt either for an open surgical approach or for a nonsurgical treatment. Differences in long term results with the two surgical options could be discussed, but differences in short term morbidity, and even in associated costs of treatment, obviously favour the minimally invasive approach [9]. Differences with nonsurgical treatments are currently a hot topic of debate [10].

Every step in a surgical technique has a reason to be. For new procedures it should be particularly important to closely follow the rules. TORS radical tonsillectomy was originally described in detail with the intention of the technique to be highly reproducible (hence, highly teachable). In our experience it certainly is. Basically, the surgical technique for radical tonsillectomy is the same as the one described more than 60 years ago. However, it is now when the transoral approach to the lateral oropaharynx has become popular. Reasons for this are surely complex but, in our view, safety and reproducibility of TORS radical tonsillectomy are at the very core of it.
